# Visual classification of allergenic pollen in iteratively reconstructed lens-less DIHM images

**DOI:** 10.1038/s41598-026-36618-8

**Published:** 2026-01-22

**Authors:** Blaž Cugmas, Eva Štruc, Mindaugas Tamosiunas, Zbigņevs Marcinkevičs, Miran Bürmen, Peter Naglič

**Affiliations:** 1 Faculty of Science and Technology, University of Latvia, Riga, LV-1004 Latvia; 2 Vetamplify SIA, Veterinary R&D Services, Riga, LV-1010 Latvia; 3 Faculty of Electrical Engineering, University of Ljubljana, Ljubljana, SI-1000 Slovenia; 4 Institute for Laser Technologies in Medicine and Metrology, University of Ulm (ILM), DE-89081 Ulm, Germany

**Keywords:** Biological techniques, Computational biology and bioinformatics, Plant sciences

## Abstract

Lens-less digital in-line holographic microscopy (DIHM) is a low-cost, wide-field imaging technique that relies on computational reconstruction to form focused images that should ideally be free of twin-image artifacts. While current DIHM-based pollen classification systems are typically automated and rely on large datasets and deep learning, our study explored whether iteratively reconstructed DIHM images using the Gerchberg–Saxton (GS) algorithm are suitable for visual classification by human experts. Two veterinary cytopathologists evaluated images of six clinically relevant pollen types, namely timothy grass, common ragweed, silver birch, common alder, olive tree, and hazel, using both lens-less DIHM and conventional optical microscopy. Classification accuracy was comparable across modalities, with DIHM achieving 95.8% and optical microscopy 96.9%. Inter-observer agreement was high (Cohen’s κ = 0.91), indicating near-perfect consistency between evaluators. Most misclassifications involved silver birch pollen, likely due to its morphological variability and overlap with common alder and hazel. These findings demonstrate that lens-less DIHM combined with iterative reconstruction enables accurate visual identification of allergenic pollen, offering a promising alternative to conventional microscopy in veterinary and other resource-limited settings.

## Introduction

Lens-less digital in-line holographic microscopy (DIHM), also known as on-chip microscopy, is a cost-effective and straightforward technique for capturing images of microscopic objects, eliminating the need for traditional optical microscope components, such as lenses and objectives. In a typical setup, the object is placed a few millimeters above the camera sensor and illuminated with a coherent or partially coherent source such as light-emitting (LED) or laser diodes (LD). The acquired in-line hologram represents the interference pattern between the unscattered (transmitted) light and the light scattered by the object. The interference pattern encodes information about the amplitude and phase of the complex wavefield at the imaging sensor, which can be backpropagated to the object plane to retrieve the object’s transmission function. A closely related lens-less technique is coherent diffraction imaging (CDI), which is widely used in X-ray and electron microscopy. Unlike DIHM, which records a near-field in-line hologram formed between the interference of the unscattered reference wave and the object-scattered wave, CDI measures only the far-field diffraction intensity, corresponding to the Fourier magnitude of the object^1^. Because the phase of the scattered wave is not recorded, CDI reconstruction relies on iterative phase retrieval using Fourier-domain constraints, whereas DIHM reconstruction involves numerical wave propagation between the object and sensor planes to recover the complex field.

The amplitude of the object’s transmission function is equivalent to the object’s brightfield image, and thus, backpropagation is necessary for the visual interpretability of the recorded hologram. However, the backpropagated in-line hologram exhibits two superimposed terms known as the “real” and “twin” images. While the “real” image is focused upon backpropagation, the “twin” image is defocused and makes it difficult to interpret the actual object’s transmission function.

Since the “real” and “twin” images cannot be separated directly by a simple backpropagation routine, computational phase-retrieval algorithms are required to recover the complex wavefront at the object plane. Two main approaches have been developed to reconstruct in-line holograms. The first approach is physics-based, employing scalar wave propagation methods, such as the angular spectrum method, for the iterative retrieval of amplitude and phase. One widely used implementation is the Gerchberg–Saxton (GS) algorithm, which alternates between the imaging and object plane while imposing known constraints in each plane. For example, the constraint in the imaging plane is the measured in-line hologram intensity itself, whereas in the object plane, the transmittance amplitude is assumed to be less than 1 (i.e., a non-amplifying object). Through iterations, this GS-based approach yields a twin-image-free reconstruction of the object’s amplitude and phase^2^.

The second reconstruction approach is based on deep learning. Rivenson et al.^3^ and Wu et al.^4^ have demonstrated that convolutional neural networks (CNNs) can be trained to perform phase recovery and holographic image reconstruction using a single input hologram. These models effectively eliminate twin-image artifacts and produce accurate images in a time-efficient manner once trained. However, such data-driven supervised training methods require a large dataset of holograms paired with corresponding actual optical microscope images, which can be cost- or labor-intensive.

Since its resolution is governed by the imaging sensor pixel size and its field of view by the sensor size, lens-less DIHM is particularly suited for examining semi-transparent particles distributed over large microscope slide areas. This makes it ideal for applications such as aerosol monitoring in environmental research^5^ and cytological sample analysis in medical diagnostics^6^. Pollen represents a particularly relevant target for DIHM because it is not only a major component of airborne aerosols but also a clinically significant allergen in both human and veterinary medicine. Consequently, there is a strong research focus on monitoring and classifying pollen^7^ for both environmental and clinical purposes.

Most existing research has relied on automated pollen classification based on DIHM (with or without lenses) and deep learning. For example, in an aerosol-monitoring study^8^, a mobile, lens-free sensor with a virtual impactor was used to concentrate particles under pulsed laser diode illumination to create a hologram and subsequently train a deep neural network to classify the pollen types (achieving accuracy of 92.9%). A compact, low-cost device^9^ based on a Raspberry Pi camera and LED illumination captured scattering patterns and used a neural network to transform these diffraction patterns into microscope-like images of the pollen. DIHM is also part of the commercial automatic pollen monitoring system (SwisensPoleno Jupiter, Swisens AG, Emmen, Switzerland)^7^. When the device was tested for pollen classification, the accuracies ranged from 90% to 97%^10,11^.

Recent studies^12–14^ have highlighted that reliable automated pollen classification often requires images captured at multiple focal planes to reveal pollen morphological features (such as spines or aperture types) at different depths. Additionally, the orientation of the pollen grain, including whether a polar or equatorial view is presented, determines which features are visible^15,16^. Consequently, automated pollen classifiers can become data-intensive and costly to develop and deploy.

In contrast, we investigated the capability of lens-less DIHM for manual, i.e., visual pollen evaluation by trained professionals, which, to our knowledge, has not been previously explored. This study relied on veterinary cytopathology experts and veterinary-relevant pollen, as pollen is a major environmental trigger of canine atopic dermatitis, the most common allergic skin disease in dogs^17,18^. We compared the classification accuracy on six pollen types using either DIHM or conventional optical microscopy. We hypothesize that (1) reconstructed lens-less DIHM images are of sufficient quality to allow for visual identification of canine-relevant allergenic pollen, and (2) the achieved classification accuracy is comparable to that of conventional optical microscopy. Our work presents an application of an under-explored imaging technique, i.e., lens-less DIHM, for straightforward and low-cost pollen detection or classification in medical or environmental samples.

## Results

**Fig. 1:**
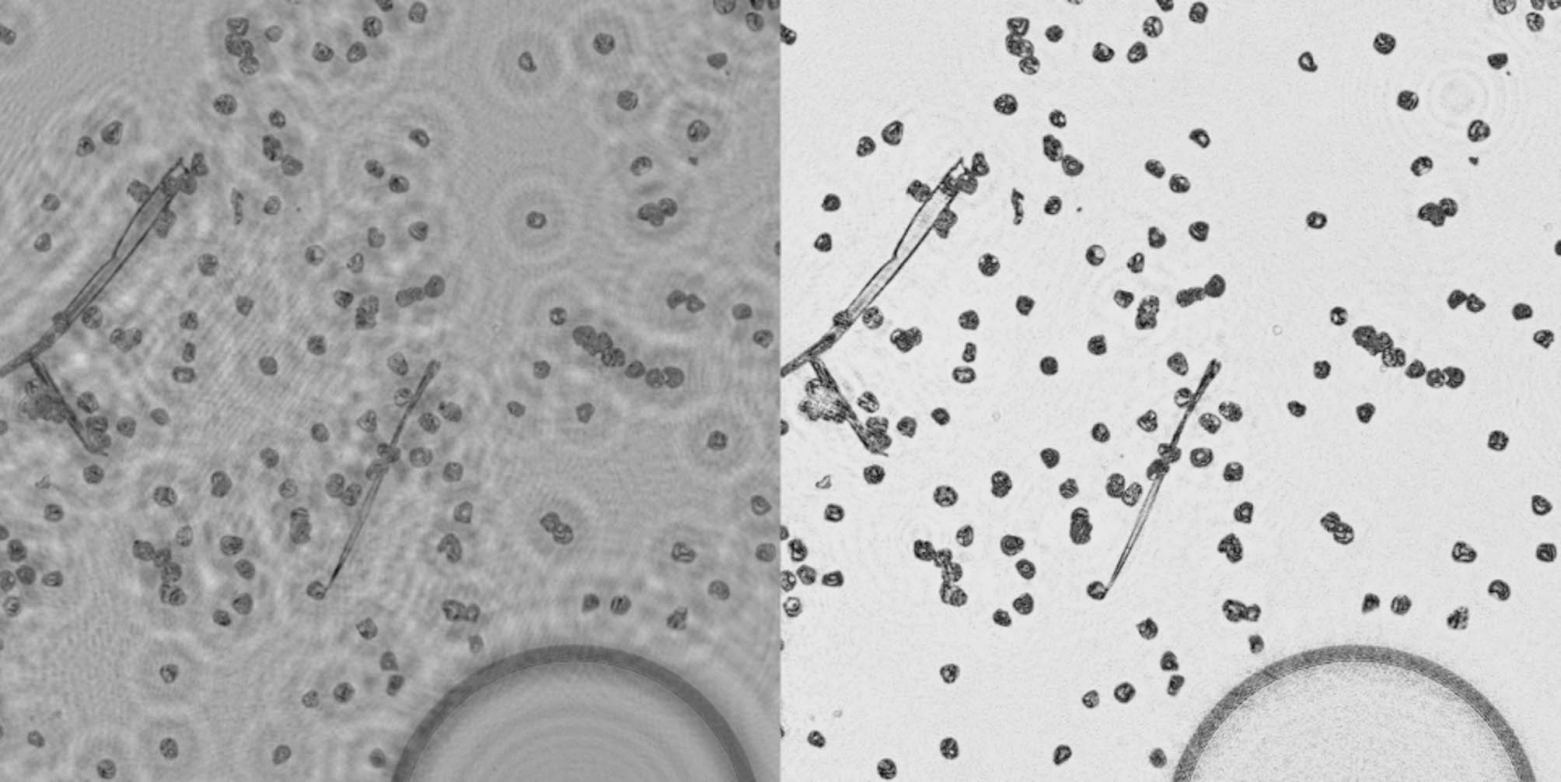
Amplitude of the transmission function in the object plane of timothy grass (*Phleum pratense*) obtained by (left) a single backpropagation and (right) iterative reconstruction of an in-line hologram (shown in Fig. 5) using the GS algorithm (200 iterations). The GS reconstruction substantially suppresses the twin-image artifact visible as the concentric interference rings in the single backpropagation result, yielding a cleaner background and clearer pollen boundaries.

Figure 1 compares two reconstruction approaches applied to an in-line hologram of pollen: (a) a single backpropagation and (b) a 200-iteration Gerchberg–Saxton (GS) phase-retrieval reconstruction. In the single-backpropagated amplitude image, each pollen grain appears in focus, but is surrounded by a diffuse halo, the result of the “twin” image artifact, which lowers contrast and impairs visual interpretation.

In contrast, the GS-reconstructed amplitude image shows a substantial suppression of the twin image, leading to a visibly cleaner background and a higher object-to-background amplitude ratio. Importantly, the fine morphological features of the pollen grains are preserved. This improved visual clarity produces an image more closely resembling conventional brightfield microscopy.

Based on these observations, the GS-reconstructed amplitude images were selected for use in the subsequent visual classification study, in which trained veterinary professionals assessed the diagnostic quality of pollen images.

**Fig. 2:**
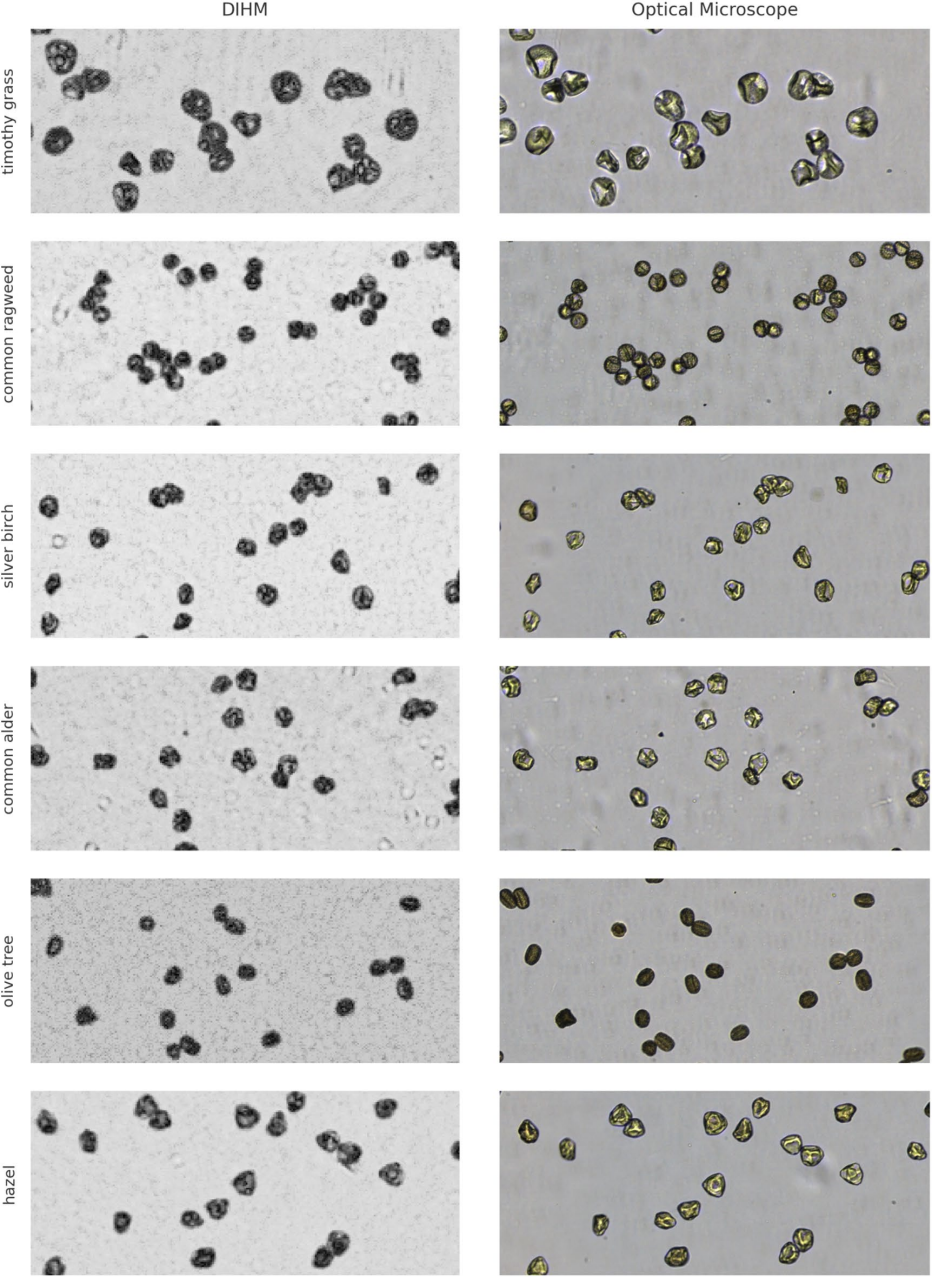
Representative images of six pollen types captured using lens-less digital in-line holographic microscopy (DIHM, left) and conventional optical microscopy (right). Six pollen were included: timothy grass (*Phleum pratense*), common ragweed (*Ambrosia artemisiifolia*), silver birch (*Betula pendula*), common alder (*Alnus glutinosa*), olive tree (*Olea europaea*), and hazel (*Corylus avellana*).

Figure 2 presents representative images of six pollen types captured using both reconstructed DIHM amplitude and conventional optical microscopy (brightfield) images, highlighting the morphological features accessible for visual assessment in each modality. 

**Table 1:** Pollen classification accuracy (in %) by two observers using optical microscopy and lens-less digital in-line holographic microscopy (DIHM). Six pollen were included: timothy grass (*Phleum pratense*), common ragweed (*Ambrosia artemisiifolia*), silver birch (*Betula pendula*), common alder (*Alnus glutinosa*), olive tree (*Olea* *europaea*), and hazel (*Corylus avellana*).

	Lens-less DIHM	Optical microscope
Expert 1	97.9	95.8
Expert 2	93.8	97.9
Average	95.8	96.9

Table 1 summarizes the visual classification accuracy achieved by two veterinary cytology experts. Overall accuracy was high with both systems: 95.8% for lens-less DIHM and 96.9% for optical microscopy. While optical microscopy showed marginally better performance, the difference amounts to the misclassification of just one additional sample. These results demonstrate that reconstructed DIHM images provide sufficient visual quality to enable expert-level classification accuracy comparable to that of traditional optical microscopy.

Observer performance was consistent across both imaging modalities. The first expert correctly classified 96.9% of the pollen samples, while the second achieved 95.8%. Inter-observer agreement, assessed using Cohen’s Kappa, yielded an overall value of 0.91, indicating almost perfect agreement. Agreement was slightly higher for classifications based on optical microscopy (κ = 0.93) compared to lens-less DIHM (κ = 0.90), but both values reflect strong consistency between experts. Furthermore, inter-modality (optical vs. DIHM) agreement was identical to inter-observer agreement (κ = 0.91). These results suggest that expert visual interpretation is highly reliable when using reconstructed DIHM images, comparable to conventional optical microscopy.

**Fig. 3:**
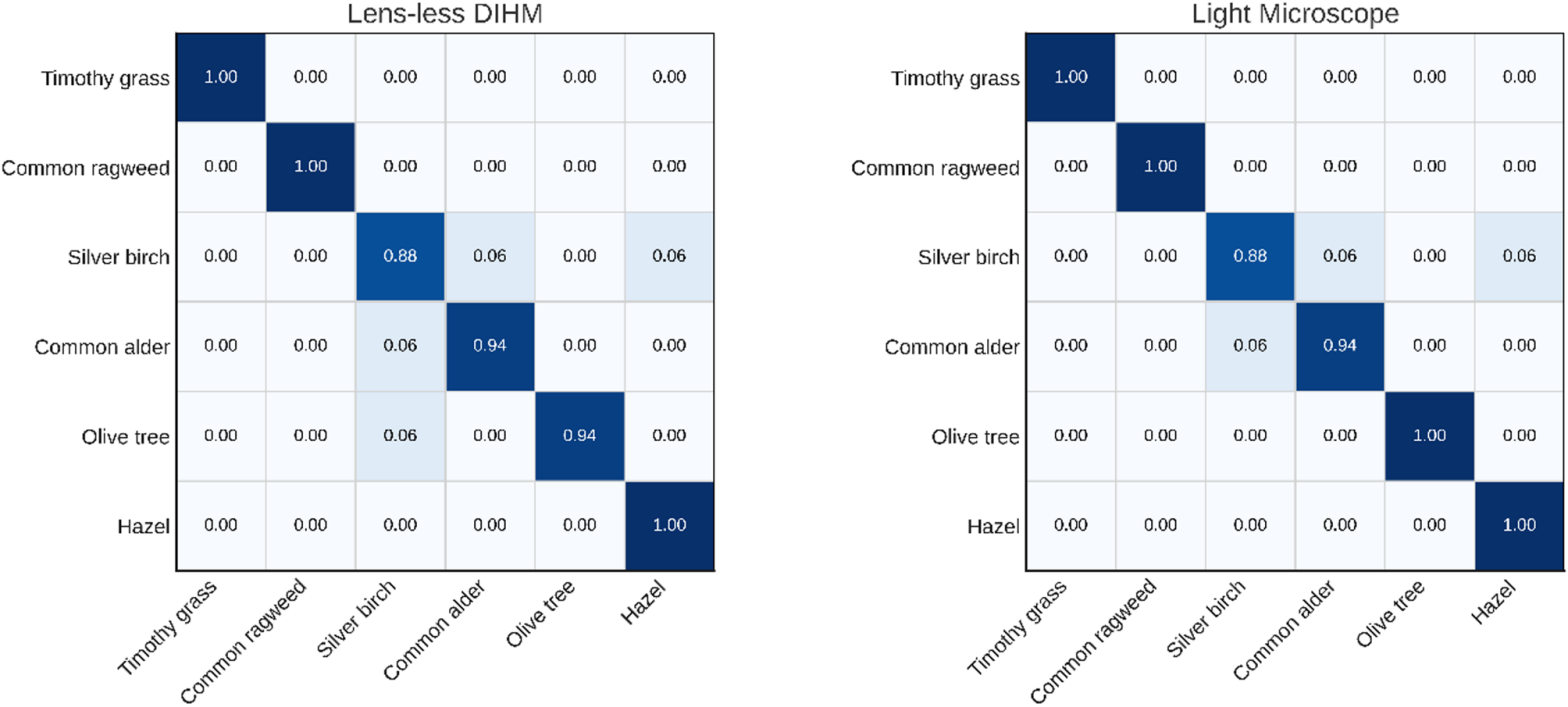
Confusion matrices showing classification accuracy of pollen using optical microscopy and digital in-line holographic microscopy (DIHM). Six pollen were included: timothy grass (*Phleum pratense*), common ragweed (*Ambrosia artemisiifolia*), silver birch (*Betula pendula*), common alder (*Alnus glutinosa*), olive tree (*Olea europaea*), and hazel (*Corylus avellana*).

Figure 3 shows normalized confusion matrices for both imaging modalities, optical microscopy and lens-less DIHM, with each row representing one of six pollen classes, based on 16 evaluations per class. For both modalities, four pollen types, namely timothy grass, common ragweed, olive tree, and hazel, were correctly classified in all cases (accuracy = 1.00), except for a single unexplained error. Pooled per-class analysis showed perfect classification performance (F1 = 1.00) for timothy grass and common ragweed. The remaining pollen types exhibited slightly lower F1-scores, 0.889 for silver birch, 0.938 for common alder, 0.984 for olive tree, and 0.970 for hazel.

**Fig. 4:**
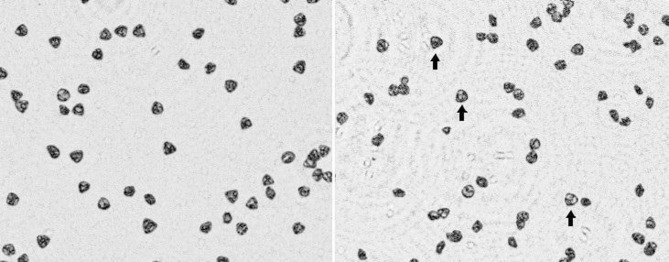
Representative lens-less DIHM images of triangular-shaped pollen. Left: hazel (*Corylus avellana*), typically exhibiting a triangular appearance. Right: silver birch (*Betula pendula*), which can occasionally include pollen grains with similar triangular morphology (arrows).

Most misclassifications (6 out of 7) occurred among silver birch, common alder, and hazel. These errors likely reflect the morphological variability of silver birch pollen, which can resemble the triangular shape of hazel or the pentagonal shape of alder (as illustrated in Fig. 4). Despite these minor overlaps, both imaging systems demonstrated strong and consistent classification performance across all pollen types, reinforcing the suitability of lens-less DIHM for expert-level visual identification.

## Discussion

Unlike previous DIHM-based pollen studies that rely on automated deep-learning classification trained on large datasets^8–10^, the present work evaluates whether GS-reconstructed DIHM images provide sufficient morphological detail for manual expert interpretation, focusing on a fundamentally different objective that cannot be directly compared to automated systems.

The expert evaluation in the present study was based solely on single-plane, iteratively reconstructed holographic images. While these reconstructions provided sufficient visual detail for high classification accuracy, they represent only a fraction of the information contained in the raw in-line holograms. A key advantage of lens-less DIHM is that a single recorded hologram captures the full complex wavefront, allowing for computational post-acquisition refocusing to any axial position. By varying the propagation distance *h* in the angular spectrum method or within iterative algorithms like Gerchberg–Saxton (GS), the same hologram can be reconstructed at multiple focal planes. This capability makes DIHM inherently volumetric and versatile, enabling the generation of multi-focus image stacks without re-imaging the sample. Such stacks can resolve ambiguities caused by overlapping or partially occluded pollen grains and provide depth-resolved morphological cues, features that have been shown to improve classification in automated systems^12–14^. Incorporating multi-plane reconstructions in future studies may further enhance expert-level visual interpretation and diagnostic confidence.

The high classification accuracy of approximately 96% across both evaluators and imaging modalities confirms that the proposed lens-less DIHM system, combined with iterative GS reconstruction, delivers sufficient resolution and visual clarity for the recognition of key pollen features. Notably, the veterinary cytopathologists participating in this study were not specifically trained in detailed palynology, i.e., the study of pollen grains. As a result, most evaluations relied on overall pollen shape rather than on fine morphological details. Experts reported that having a clear and well-defined pollen outline was crucial for their decision-making, favoring the higher-contrast, twin-image-suppressed reconstructions over single-backpropagation images, which exhibited halo artifacts and reduced edge definition. In practice, classification was primarily based on distinct shape features, with secondary cues drawn from visible spines (e.g., in common ragweed) and apertures (e.g., in hazel pollen). However, larger-scale morphological features such as overall shape and prominent spines were reliably visible in DIHM images, while finer features such as apertures and pores generally exhibited reduced contrast compared to optical microscopy. Anyway, high-quality edge definition in reconstructed DIHM images is sufficient for reliable visual classification of basic pollen, even without deep morphological training.

Moreover, the comparable classification performance between lens-less DIHM and conventional optical microscopy suggests that DIHM can replicate the diagnostic capability of optical microscopy, while offering notable advantages in terms of simplicity, cost-efficiency, and portability. Our results align with previous studies on automated pollen classification using holographic microscopy, which reported accuracies ranging from 90% to 97%^8,10,11^ across similar numbers of pollen types (typically 6–8). The consistency in both classification accuracy and variety of pollen types reinforces the validity and generalizability of our approach, even when applied to manual expert evaluation rather than machine learning models.

Some pollen types were consistently easier to identify due to their distinctive morphology. For example, olive tree pollen resembled coffee beans, while common ragweed appeared as uniformly round grains with prominent spike-like surface features. Grass pollen was also relatively recognizable, and the presence of plant debris on the slides (Fig. 1) may have provided contextual clues that aided classification. In contrast, the most frequent misclassifications involved silver birch, whose highly variable morphology led to confusion with common alder and hazel (Figs. 3 and 4), which made it harder for experts to consistently identify defining features. These observations suggest that classification accuracy may depend not only on image quality but also on whether the evaluator is able to locate characteristic, representative pollen grains within the field of view. This challenge mirrors a known limitation in automated pollen monitoring systems, where morphological overlap and orientation variability led to similar misclassifications^15,16^.

Although inter-observer agreement was high (κ = 0.91), it fell slightly short of perfect agreement, indicating occasional differences in expert interpretation. Interestingly, only one misclassification was shared between both evaluators: a silver birch pollen grain that was mistakenly identified as hazel. This particular error is understandable given the morphological similarities and overlap between these two taxa, as discussed earlier.

Visual evaluation of pollen using lens-less DIHM holds significant promise for resource-limited settings, such as veterinary medicine, where access to advanced laboratory equipment is often restricted. In dogs, pollen is a major environmental trigger for canine atopic dermatitis (CAD), the most common allergic skin disease, affecting up to 15% of the canine population worldwide^17^. CAD is characterized by chronic itching, inflammation, and recurrent skin infections, which substantially impact the quality of life for both animals and their owners. Although therapies such as immunotherapy and symptomatic treatment can help manage the condition, the underlying pathogenesis remains incompletely understood^18,19^, particularly regarding host–environment interactions. Notably, a recent study reported no clear correlation between airborne pollen levels and pruritus severity in atopic dogs^20^, highlighting the need for more direct and individualized pollen monitoring methods^21^. Previous research has attempted to identify pollen grains on animal coats or in fecal samples using conventional optical microscopy^22,23^. Thus, lens-less DIHM could provide a practical and scalable alternative for such applications without the need for expensive optical systems.

Despite the promising results, several limitations should be acknowledged. First, although the dataset was intentionally balanced with an equal number of samples per pollen type, this design choice may have introduced a subtle bias. Given the small sample size, evaluators might have inferred which classes were underrepresented, potentially influencing their decisions. Second, the pollen concentration per slide was relatively high, often containing hundreds of grains. This likely increased the chance of encountering representative pollen, possibly enhancing classification performance compared to more challenging real-world scenarios. In clinical veterinary settings, impression samples taken from skin or fur may contain far fewer grains (as few as ~ 20), some of which may be damaged, partially obscured, or poorly oriented. Moreover, unlike routine microscopic analysis, where grains can be physically repositioned, our samples were fixed in silicone, preventing any manipulation. However, the higher number of visible grains in our setup may have partially offset this limitation by presenting multiple orientations on a single slide. Finally, plant debris was present in the grass pollen samples, but not in other types. This may have unintentionally aided recognition by providing contextual clues unrelated to the pollen morphology itself, representing another potential source of bias.

## Methods

### DIHM setup

**Fig. 5:**
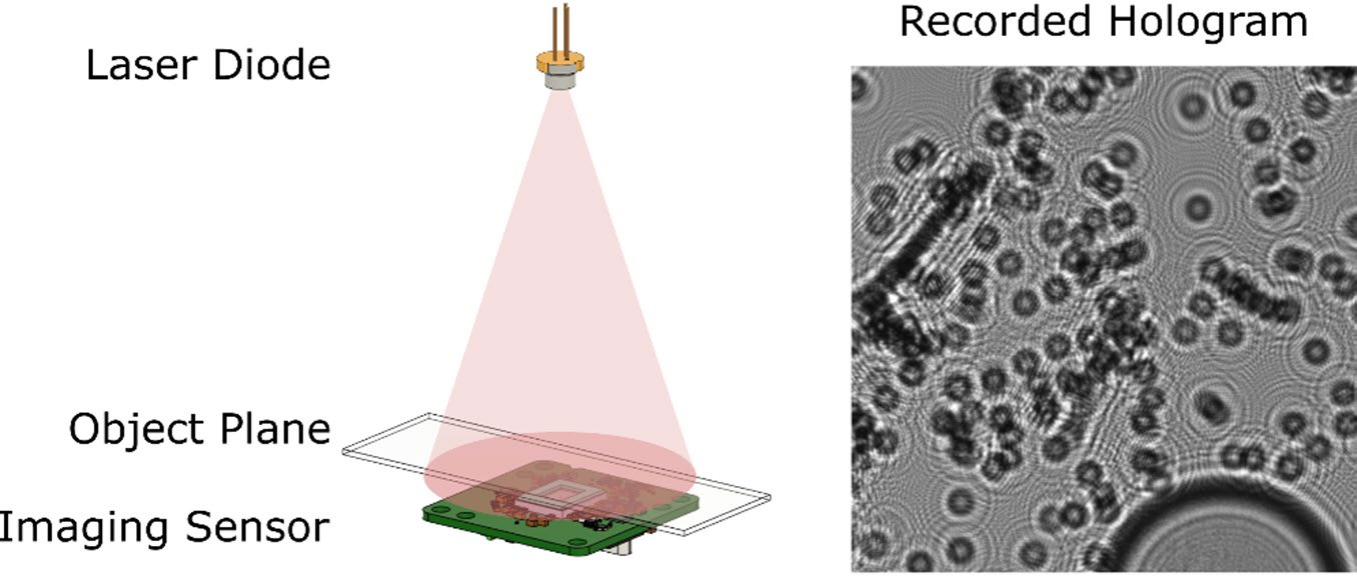
Experimental setup of the DIHM and an example of a recorded hologram reconstructed in Fig. 1.

A custom-built, lens-less digital in-line holographic microscope (DIHM) was used to acquire holograms of pollen samples. The imaging system employed a board-level, no-mount monochrome camera (model daA3840-45 μm, Basler AG, Ahrensburg, Germany), equipped with a Sony IMX334 sensor. The sensor has a physical size of 7.68 × 4.32 mm² and a pixel size of 2.0 × 2.0 μm². A custom 3D-printed slide holder was mounted above the camera on an XY translation stage, enabling precise positioning of samples. Standard 1 mm-thick glass microscope slides were used, resulting in an object-to-sensor distance of approximately 3–4 mm. Illumination was provided by a 658 nm red laser diode (L658P040, Thorlabs, Newton, NJ, USA), mounted in a TCLDM9 laser diode mount (Thorlabs) and positioned approximately 20 cm above the sensor. This configuration ensured that the incident wavefront could be treated as a quasi-plane wave, providing the spatial coherence necessary for in-line holography. The long distance between the laser and sample, combined with the small emission area of the diode, maintained a high degree of spatial coherence across the field of view. The schematic of the experimental setup is provided in Fig. 5.

## Pollen samples

The pollen samples used in this study were obtained from Nextmune AB (Stockholm, Sweden), which provided individual vials containing pure, dry pollen of each species. Six pollen types were included in this study based on their clinical relevance to canine atopic dermatitis: timothy grass (*Phleum pratense*), common ragweed (*Ambrosia artemisiifolia*), silver birch (*Betula pendula*), common alder (*Alnus glutinosa*), olive tree (*Olea europaea*), and hazel (*Corylus avellana*). Each pollen type was presented on 10 microscope slides, resulting in a total of 60 slides.

To prepare the slides, pollen was embedded in a silicone mounting medium (PlatSil^®^ SiliGlass, Polytek Development Corp., Easton, PA, USA) and sealed with coverslips. For each preparation, 1.5 g of Phase A and 1.5 g of Phase B silicone were measured separately. 40–50 mg of dry pollen was mixed into one of the phases, depending on the pollen type. Then, 400 µL from each phase was combined and thoroughly mixed. From this mixture, 30–40 µL was pipetted onto each slide, followed by placement of a coverslip. Slides were left to cure at room temperature until the silicone solidified.

In some cases, further dilution was required to ensure comparable visual pollen densities across slides. This was achieved by adding 40–80 µL of silicone (either Phase A or B) to the base mixture before pipetting. For each pollen type, 10 slides with different dilution levels were prepared to achieve visually balanced concentrations across all samples.

## Reconstruction of the in-line holograms

To reconstruct the object-plane amplitude from a raw in-line hologram, we first applied the angular spectrum method, a scalar diffraction model that represents wave propagation between two planes as a superposition of plane waves traveling at different angles^24^. This single-step backpropagation yields a complex wavefield in the object plane. However, it still includes the defocused “twin” image artifact, a well-known limitation of in-line holography that degrades visual clarity and contrast.

**Fig. 6:**
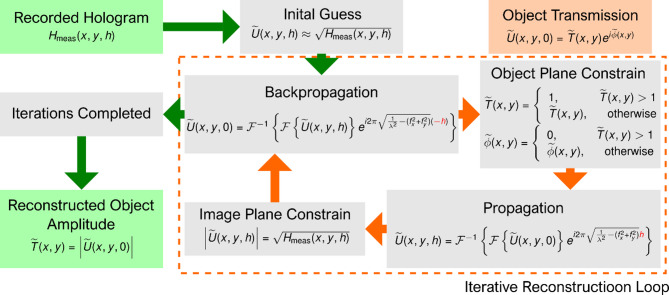
Flowchart for iterative reconstruction of in-line holographic images.

To address this limitation, we additionally implemented an iterative phase-retrieval approach based on the Gerchberg–Saxton (GS) algorithm, which aims to eliminate the twin image by alternately propagating the complex wavefield between the object and imaging planes while imposing physical constraints in each domain. The method follows the implementation described by Latychevskaia^2^, and a summary of the reconstruction workflow is presented in Fig. 6.

The final output of the iterative GS reconstruction is a twin-image-suppressed, high-contrast amplitude image, which more closely resembles a brightfield optical microscopy image and was used for all expert evaluations in this study.

The reconstruction procedure began with the raw hologram $\:{H}_{meas}\left(x,y,h\right)\:$, which represents the intensity at the imaging plane located at a distance *h* from the object. The hologram was first normalized to a background value, and its square root was used to form the initial guess of the complex wavefield $\:\stackrel{\sim}{U}\left(x,y,h\right)$ at the imaging plane:

$$\:\stackrel{\sim}{U}\left(x,y,h\right)=\sqrt{{H}_{meas}(x,y,z)}$$

Accurate estimation of the propagation distance *h* is critical, as it significantly affects reconstruction quality. In this study, we determined *h* by performing a single backpropagation and manually selecting the axial distance at which the pollen grains appeared visually sharpest. While this distance could, in principle, be measured experimentally, micrometer-level precision is difficult to achieve reliably in practice. On average, the refocusing distance *h* between the imaging and object planes for all pollen types was 2546 μm (standard deviation 54 μm).

After determining *h*, we applied an iterative phase-retrieval algorithm based on the GS method, propagating the complex wavefield back and forth between the imaging and object planes. At each iteration, constraints were applied in both planes:

- In the object plane, we imposed an amplitude constraint assuming that the object cannot amplify light. The transmission amplitude $\:\stackrel{\sim}{T}\left(x,y\right)$ was clipped such that values exceeding 1 were set to 1, and the corresponding phase values $\:\stackrel{\sim}{\varphi\:}\left(x,y\right)$ were reset to zero.
- In the imaging plane, the amplitude of $\:\stackrel{\sim}{U}\left(x,y,h\right)$ was updated at each iteration using the measured hologram intensity:

$$\:\stackrel{\sim}{|U}\left(x,y,h\right)|\leftarrow\:\sqrt{{H}_{meas}(x,y,z)}$$

The algorithm was run for 200 iterations, which was sufficient to achieve convergence in all cases. The final result is the reconstructed object transmission function, comprising the amplitude $\:\stackrel{\sim}{T}\left(x,y\right)$, which corresponds to a brightfield-like image, and the phase $\:\stackrel{\sim}{\varphi\:}\left(x,y\right)$, which was not further analyzed in this study. Only the amplitude reconstructions were used for visual evaluation by the experts.

### Evaluation of pollen samples

In parallel with holographic imaging, the same areas of each microscope slide were scanned using a conventional inverted microscope (Nikon Ti2-E) equipped with a 10×/0.3 NA objective (Nikon CFI Plan Fluor) and a Basler ACE acA1920-155 μm color camera mounted on the side port via a C-DA C-Mount adapter. The resulting uncompressed color images were saved in TIFF format at a resolution of 15,876 × 7,783 pixels, with 24-bit color depth and 96 DPI, resulting in a file size of approximately 360 MB per image.

By comparison, the uncompressed holographic images were also saved as TIFF files, with a resolution of 3,840 × 2,160 pixels, a 12-bit depth, and a file size of approximately 16 MB per image.

Two evaluators, both holding Doctor of Veterinary Medicine (DVM) degrees with specialized training in veterinary cytopathology, participated in the visual classification study. The evaluation was conducted over two consecutive days, with each imaging modality assessed on a separate day. To minimize order bias, one evaluator began with the DIHM images, while the other started with optical microscopy images. On the second day, the evaluators switched modalities.

Each evaluator was presented with 60 images per modality, consisting of 48 test images and 12 training images (two per pollen type). Training images were randomly selected from the full dataset and were available for continuous reference throughout the session. The 48 test images were shown in a randomized order, which was unique for each modality. During evaluation, images were displayed one at a time, and evaluators were required to classify the pollen type without the option to revisit previous images.

No specialized image analysis tools (e.g., segmentation, size measurement) were used. All evaluations were conducted using the default Windows 10 Photos application, simulating standard diagnostic workflows in microscopy, where visual inspection is the primary diagnostic method.

Classification accuracy was calculated as the number of correctly classified test images divided by the total number of test images (*n* = 48), computed separately for each evaluator and modality. Inter-observer/-modality agreements were assessed using Cohen’s Kappa coefficient, which accounts for chance-level agreement between two raters. A Kappa value of 1.0 indicates perfect agreement, while 0.0 indicates agreement expected by chance.

All statistical analyses were performed in Python 3.12.2 using the Scikit-learn library (version 1.7.0) for Kappa calculation (cohen_kappa_score) and Matplotlib (version 3.6.3) for plotting confusion matrices. Analyses were run in the Spyder IDE (version 6.0.7).

## Data Availability

The datasets generated during and/or analysed during the current study are available from the corresponding author on reasonable request.
